# The Diagnosis Is in the Smear: A Case and Review of Spur Cell Anemia in Cirrhosis

**DOI:** 10.1155/2021/8883335

**Published:** 2021-03-26

**Authors:** Gabriella A. Raffa, Diana M. Byrnes, John J. Byrnes

**Affiliations:** ^1^Internal Medicine Residency Program, Jackson Memorial Hospital, Miami, FL, USA; ^2^Hematology/Oncology Fellowship Program, Jackson Memorial Hospital, Miami, FL, USA; ^3^Department of Medicine, Division of Hematology, University of Miami, Miami, FL, USA

## Abstract

The etiology of anemia in liver cirrhosis is multifactorial; one less recognized cause is hemolytic anemia due to spur cells, known as spur cell anemia. We present the case of a 57-year-old woman with alcoholic cirrhosis who presented with symptomatic macrocytic anemia with a hemoglobin level of 7.4 g/dL and signs of decompensated liver disease. Notably, she had no signs of overt bleeding. Further workup was consistent with hemolysis, with peripheral smear demonstrating spur cells. The patient was treated with both steroids and IVIG, although she eventually expired. The characteristic morphology of spur cells is due to alteration of the lipid composition of the erythrocyte membrane, changing its shape and leading to splenic sequestration and destruction. Characteristic of this disorder is an increased ratio of cholesterol to phospholipid on the membrane, as well as low levels of apolipoproteins and low- and high-density lipoproteins. The presence of spur cells is an indicator of poor prognosis and high risk of mortality. Currently, the only definitive cure is liver transplantation. There is a paucity of literature on the prevalence of this phenomenon and even less about treatment. This case highlights the importance of recognition of spur cell anemia as a cause of anemia in cirrhosis as well as the importance of the peripheral smear in the diagnostic workup. Early recognition can lead to avoidance of unnecessary procedures. Further research is needed to elucidate the true prevalence of spur cell anemia and examine further treatment options.

## 1. Introduction

The systemic sequelae of liver cirrhosis are vast and consist of hematological abnormalities, including anemia. The etiology of anemia in cirrhotic patients is multifactorial and can be secondary to red blood cell destruction by splenic sequestration and hypersplenism, bone marrow suppression, vitamin deficiencies, and occult or chronic bleeding from varices or gastropathy [1]. Less commonly generated in the differential, although unmistakable on peripheral smear, is that of spur cell anemia (SCA) which induces a hemolytic anemia in patients with liver disease. SCA is associated with high mortality and treatment options are limited. Here, we report a case of SCA in the setting of alcoholic liver cirrhosis.

## 2. Case Report

**Figure 1:**
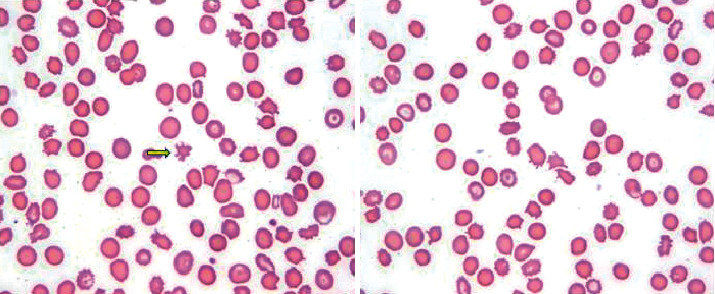
Peripheral smear using 100x objective lens demonstrating numerous acanthocytes (spur cells), spherocytes, target cells, and occasional burr cells. An example of a spur cell is identified with a yellow arrow.

We present the case of a 57-year-old Hispanic woman with a past medical history of alcoholic cirrhosis (MELD-Na 30 on admission, Child-Pugh Class C) complicated by Grade I esophageal varices and portal hypertensive gastropathy, as well as recently diagnosed stage I colorectal adenocarcinoma (treatment naïve), who presented to the hospital with a one-week history of fatigue, dyspnea, and dizziness. On admission, the patient was noted to be hypotensive with a blood pressure of 89/48, afebrile with a temperature of 98.4 °F, and tachycardic with a heart rate of 100 beats per minute. Physical exam was notable for appearance of weakness, although the patient was awake, alert, and oriented, with scleral icterus, jaundice, splenomegaly, ascites, and mild abdominal distension. Laboratory exams in the initial days of presentation are noted in Table 1 and are significant for macrocytic anemia with a hemoglobin of 7.4 g/dL and a mean corpuscular volume of 109.4 fL. Studies were consistent with hemolysis, with an elevated LDH of 263 U/L, indirect hyperbilirubinemia of 9.2 mg/dL, undetectable haptoglobin (<10 mg/dL), and elevated reticulocyte count of 16.1% (Table 1). Her macrocytosis was most likely secondary to reticulocytosis due to hemolysis, although workup of vitamin deficiencies was initiated, demonstrating normal folate levels and elevated vitamin B12 levels, likely in the setting of severe liver disease [2, 3]. Peripheral smear demonstrated mostly acanthocytes (spur cells), spherocytes, rare schistocytes, target cells, and few burr cells (Figure 1). The patient was resuscitated with packed red blood cells and fluids and briefly placed on vasopressors. Notably, the patient had no clinical evidence of upper or lower gastrointestinal bleeding. For this reason, the patient did not undergo endoscopy or colonoscopy during her admission.

On further workup of her hemolytic anemia, the patient was tested for paroxysmal nocturnal hemoglobinuria with FLAER, which was negative, as was direct Coombs testing. Of note, three months prior to this presentation, during a previous admission, the patient had a direct Coombs test that was positive for IgG antibody; elution testing revealed a warm autoantibody. It was speculated that perhaps the patient had a low level of antibodies, not detectable during this admission, and that she perhaps had warm autoimmune hemolytic anemia (WAIHA). However, during her previous admission, when she tested positive, she had also received a blood transfusion at that time which could have contributed to a false positive. Additional workup included CT of the abdomen and pelvis to assess for lymphadenopathy given the association of lymphoproliferative disorders and WAIHA, which was negative for lymphadenopathy although remarkable for splenomegaly of 14.4 cm. She was initially treated for WAIHA with steroids at 1 mg/kg for three days, increased to 1.5 mg/kg for five days with no clinical response in terms of her hemolysis panel; therefore, a steroid taper was started. Due to the refractory nature, she was treated with IVIG 1 mg/kg for two days. The new leading diagnosis was SCA because of findings on peripheral smear and lack of response to steroid therapy. Lipid levels, including apolipoprotein levels, were obtained, given the association with SCA and defects in lipid metabolism, and were notable for decreased total cholesterol, LDL, and apolipoproteins AI and B (Table 2). After receiving IVIG, her hemolysis panel was unchanged, except for the indirect bilirubin which continued to worsen (Table 3). Unfortunately, several days later, the patient continued to decompensate and expired from acute respiratory distress syndrome secondary to pneumonia.

## 3. Discussion

Acanthocytes, or spur cells, consist of characteristic spicules seen on peripheral smear. The mechanism is thought to be alteration of lipid metabolism on the erythrocyte membrane, leading to the characteristic morphology that has a propensity for hemolysis. As delineated previously, the etiologies of anemia in liver disease are vast. Portal hypertension can lead to enlargement of the spleen (as seen in this patient), causing sequestration and destruction of red and white blood cells as well as platelets. Another important consideration is disseminated intravascular coagulation (DIC), which has a number of causes, including cirrhosis and malignancy, and should be on the differential in any patient with elevation of coagulation tests and thrombocytopenia. Although this patient's normal fibrinogen, rare schistocytes on smear, and chronically elevated coagulation studies do not wholly support acute DIC, the chronic form is an important concern and could exacerbate an occult bleed from varices or gastropathy, leading to anemia. Specifically, the differential of hemolytic anemia in alcoholics with liver disease, in addition to SCA, includes a pathology known as Zieve syndrome, a condition characterized by hemolytic anemia, jaundice, and transient hyperlipidemia. The pathophysiology is attributed to alcohol toxicity to the erythrocyte membranes, leading to hemolysis and release of lipid into the circulation [4]; spur cells are usually not seen on peripheral smear [5]. Zieve syndrome is seen in milder liver disease, and the prognosis is markedly different: cessation of alcohol use usually resolves the anemia and other sequelae.

The liver plays a key role in the metabolism and homeostasis of lipids, and thus, liver dysfunction has modification of lipid homeostasis as a known side effect. In alcoholic cirrhosis, fat accumulation in the liver contributes to this dysfunction [6]. It has been observed that in liver disease, the erythrocyte membrane has an increased ratio of cholesterol to phospholipid [7, 8]. This is in part due to the fact that in the setting of liver disease, the activity of serum lecithin cholesterol acyltransferase, an enzyme which converts free cholesterol into cholesterol ester, is decreased, and consequently, the free cholesterol is elevated. This cholesterol accumulation increases erythrocyte surface area, altering its shape [7, 9], leading to splenic sequestration and subsequent destruction [10].

Other lipid profile abnormalities have been observed in liver disease. For example, Duhamel et al. noted that significantly low levels of apolipoprotein A (apoA)-II, high-density lipoprotein (HDL) III, and low-density lipoprotein (LDL) appeared characteristic of alcoholic cirrhotics with spur cells, with a trend toward lower apolipoprotein B (apoB) levels [11]. Although we do not have a baseline apoB for our patient, her level was on the low end of normal (40 mg/dL) when measured during admission. Cicognani et al. demonstrated an inverse relationship between levels of LDL, HDL, and total serum cholesterol level with severity of liver disease [12]. Notably, decreased levels of Apo-AI, as seen in our patient, seem to be a marker of fibrosis in alcoholic patients, potentially secondary to binding to fibronectin [6]. Indeed, studies have demonstrated a worsening lipid profile with increasing severity of liver disease [13, 14]. It is worth noting tht five months prior to presentation, the patient had a MELD-Na of 25 and was Child-Pugh Class B (compared to MELD-Na 30 with Child-Pugh Class C on presentation) with the lipid panel as seen in Table 2, with a trend toward decreasing total cholesterol and LDL (albeit with an increase in HDL). The presence of spur cell anemia has been associated with poor prognosis in cirrhotic patients; one study reported a median survival of 1.9 months in patients with >5% spur cells [15]. Similarly, a study by Vassiliadis et al. observed that presence of >5% spur cells correlated with lower 3-month survival rates [5].

Other sequelae of SCA include a prolonged INR. This elevation is in part secondary to the diminished synthesis of coagulation factors seen in liver disease and often concomitant vitamin K deficiency that is seen with malnutrition and alcoholism, although in the setting of SCA, it is also postulated to be due to the alteration of the phospholipid components of thromboplastin reagents [5, 16]. In addition, it has been reported that platelets also have alteration of membrane lipids, warping the meshwork used in clot formation [16]. However, the additional cholesterol on platelet membranes appears to induce platelet aggregation [17]. Sundaram et al. hypothesized that this hypercoagulable state may offset the bleeding diathesis that would otherwise be expected in the setting of a pronounced coagulopathy. Both the Child-Pugh and MELD scores, used as an estimation of cirrhosis severity and for liver transplant allocation, respectively, utilize the INR in their calculation. Given the association of SCA with severe liver disease, the effect of the pathologic mechanisms of SCA on the already present coagulopathy may be serendipitous, as this would increase the MELD score and the likelihood for a transplant.

There is a paucity of literature on the true prevalence of spur cell anemia, and even less so on treatment modalities. The only definitive cure for spur cell anemia is liver transplant, and complete reversal of anemia has been reported after transplantation [18]. Unfortunately, our patient was not a candidate for liver transplant given her active workup for colorectal adenocarcinoma. Plasmapheresis has historically been attempted. One study comparing lipid profiles before and after plasmapheresis demonstrated an increase in concentrations of total cholesterol, phospholipids, and apo-AI after treatment, although low levels of lipoprotein (a), LDL, and apo-AII persisted. Reticulocyte counts and hemoglobin levels were also noted to improve, although other markers of hemolysis were constant [19]. A case of spur cell anemia has been reported with successful improvement of hemolysis panel, decrease in number of spur cells, and decrease in the amount of free cholesterol in the blood with combination therapy of flunarizine, pentoxifylline, and cholestyramine, with remission achieved for almost a year with cholestyramine alone [20].

This case serves as a review of spur cell anemia as a cause of hemolytic anemia in advanced cirrhosis and highlights the importance of the peripheral smear in its diagnosis. Clinician awareness of the differential of hemolytic anemia in alcoholic liver disease should prompt ordering of a lipid profile, which is otherwise not normally ordered in an anemic cirrhotic patient. Early recognition could not only lead to early treatment but also prevent unnecessary procedures, namely, an elaborate endoscopic search for a gastrointestinal bleed. In addition, this report details the lack of efficacy of steroids and IVIG in treating this form of hemolytic anemia. Further research is needed to define treatment options and more accurately delineate the true prevalence of this etiology of anemia.

## Figures and Tables

**Table 1:** Laboratory data from admission.

*Complete blood count*
WBC (4.0–10.5 10^3^/*μ*L)	9.4
Hgb (11.1–14.6 g/dL)	7.4
Hct (33.2–43.4%)	22.1
MCV (80–100 fL)	109.4
Plt (140–400 10^3^/*μ*L)	51

*Serum chemistries*
Sodium (135–146 mmol/L)	131
Potassium (3.5–5.5 mmol/L)	5.0
Chloride (98–110 mmol/L)	101
Bicarbonate (19–34 mmol/L)	20
BUN (6–20 mg/dL)	16
Creatinine (0.4–1.1 mg/dL)	0.58
Total bilirubin (0–1.2 mg/dL)	15.4
Direct bilirubin (0.0–0.3 mg/dL)	6.2
Total protein (6.1–8.1 g/dL)	4.5
Albumin (3.5–5.2 g/dL)	2.3
AST (10–40 U/L)	48
ALT (0–33 U/L)	33
Alkaline phosphatase (35–130 U/L)	118

*Anemia workup*
Vitamin B12 (232–1,245 pg/mL)	1,925
Folate (>7.3 ng/mL)	11.1
Iron (37–145 mcg/dL)	148
TIBC (200–400 mcg/dL)	220
Ferritin (13–150 mg/mL)	203
%transferrin sat. (14–50%)	67.3
Reticulocyte%	16.1
LDH (135–214 U/L)	263
Indirect bilirubin (calculated)	9.2
Haptoglobin (30–200 mg/dL)	<10

*PNH with FLAER (GPI-linked CD59) negative*
Direct Coombs	Negative

*Coagulation studies*
PTT (23.4–36.0 sec)	46.1
PT (12.0–14.5 sec)	28.5
INR (0.88–1.12)	2.7
Fibrinogen (164–498 mg/dL)	206

Note that iron studies were obtained after transfusion.

**Table 2:** Comparison of lipid panel before admission and during hospitalization.

Lipid Panel	5 months prior	Current
Cholesterol (<240 mg/dL)	257	132
Triglycerides (<150 mg/dL)	76	74
HDL (>49 mg/dL)	46	59
LDL (<130 mg/dL)	193	59
Apolipoprotein A1 (>125 mg/dL)	71	
Apolipoprotein B (<90 mg/dL)	40	

**Table 3:** Hemolysis panel during hospitalization.

	Day 2	Pre-steroids (Day 6)	Pre-IVIG^*∗*^ (Day 13)	Post-IVIG (Day 15)
Hgb	7.4	9.0	7.4	7.0
Haptoglobin	<10	<10	<10	<10
Indirect bili	9.2	8	11.4	17.3
LDH	263	393	285	292

^*∗*^Patient was initiated on steroids on Day 6 at 1 mg/kg for three days, increased to 1.5 mg/kg for five days (day 8 through 13) with no clinical response, and was subsequently placed on a taper which was continued until her death.

## Data Availability

The data used to support the findings of this study are included within the article.
